# Validation of the CAchexia SCOre (CASCO). Staging Cancer Patients: The Use of miniCASCO as a Simplified Tool

**DOI:** 10.3389/fphys.2017.00092

**Published:** 2017-02-17

**Authors:** Josep M. Argilés, Angelica Betancourt, Joan Guàrdia-Olmos, Maribel Peró-Cebollero, Francisco J. López-Soriano, Clelia Madeddu, Roberto Serpe, Sílvia Busquets

**Affiliations:** ^1^Cancer Research Group, Department of Biochemistry and Molecular Biomedicine, Faculty of Biology, University of BarcelonaBarcelona, Spain; ^2^Institute of Biomedicine (IBUB)Barcelona, Spain; ^3^Advanced Statistical Data Analysis Applied to Psychology, Faculty de Psicology, University of BarcelonaBarcelona, Spain; ^4^Institute for Research on the Brain, Cognition and BehaviourBarcelona, Spain; ^5^Department of Medical Oncology, University of CagliariCagliari, Italy

**Keywords:** cachexia, wasting, anorexia, weight loss, physical performance, quality of life, classification, score

## Abstract

The CAchexia SCOre (CASCO) was described as a tool for the staging of cachectic cancer patients. The aim of this study is to show the metric properties of CASCO in order to classify cachectic cancer patients into three different groups, which are associated with a numerical scoring. The final aim was to clinically validate CASCO for its use in the classification of cachectic cancer patients in clinical practice. We carried out a case -control study that enrolled prospectively 186 cancer patients and 95 age-matched controls. The score includes five components: (1) body weight loss and composition, (2) inflammation/metabolic disturbances/immunosuppression, (3) physical performance, (4) anorexia, and (5) quality of life. The present study provides clinical validation for the use of the score. In order to show the metric properties of CASCO, three different groups of cachectic cancer patients were established according to the results obtained with the statistical approach used: mild cachexia (15 ≤ × ≤ 28), moderate cachexia (29 ≤ × ≤ 46), and severe cachexia (47 ≤ × ≤ 100). In addition, a simplified version of CASCO, MiniCASCO (MCASCO), was also presented and it contributes as a valid and easy-to-use tool for cachexia staging. Significant statistically correlations were found between CASCO and other validated indexes such as Eastern Cooperative Oncology Group (ECOG) and the subjective diagnosis of cachexia by specialized oncologists. A very significant estimated correlation between CASCO and MCASCO was found that suggests that MCASCO might constitute an easy and valid tool for the staging of the cachectic cancer patients. CASCO and MCASCO provide a new tool for the quantitative staging of cachectic cancer patients with a clear advantage over previous classifications.

## Introduction

Cancer cachexia is a syndrome present in a large number of cancer patients that results in body weight loss, inflammation, reduced physical performance, and decreased quality of life (Evans et al., 2008; Muscaritoli et al., 2010; Fearon et al., 2011; Cederholm et al., 2016). For instance, according to Evans et al: “cachexia, is a complex metabolic syndrome associated with underlying illness and characterized by loss of muscle with or without loss of fat mass. The prominent clinical feature of cachexia is weight loss in adults (corrected for fluid retention) or growth failure in children (excluding endocrine disorders). Anorexia, inflammation, insulin resistance, and increased muscle protein breakdown are frequently associated with cachexia. Cachexia is distinct from starvation, age-related loss of muscle mass, primary depression, malabsorption, and hyperthyroidism and is associated with increased morbidity” and it can be classified using the following criteria: (a) if the patient has a 5% loss of edema-free body weight during the previous 12 months or less and (b) the presence of at least three of following five characteristics: decreased muscle strength, fatigue, anorexia, low fat-free mass index, or abnormal biochemistry (Evans et al., 2008). Although several definitions exist, they share common features (Argilés et al., 2010). In spite of the fact that, in addition to definition, diagnostic criteria have been established (Evans et al., 2008), only few studies deal with cachexia staging and classification of patients (Bozzetti and Mariani, 2009; Gabison et al., 2010). From this point of view, Fearon et al. (2011) have established a classification of the syndrome based on inflammation and body weight loss. Indeed, according to this study: “Severity can be classified according to the degree of depletion of energy stores and body protein (lean body mass) in combination with the degree of on going weight loss. Assessment for classification and clinical management should include the following domains: anorexia or reduced food intake, catabolic drive, muscle mass, and strength, functional, and psychosocial impairment.” However, this study only allows a qualitative classification of the different cachectic patients, such as precachexia, cachexia, and refractory cachexia. A couple of recent papers also proposed a grading system: (1) incorporating the independent prognostic significance of both BMI and percentage of weight loss (Martin et al., 2015) or (2) according to changes on biochemistry (high C-reactive protein or leukocytes, or hypoalbuminemia, or anemia), food intake, weight loss, and performance status (Vigano et al., 2016). CASCO was designed to fulfill the gap of a numerical classification system and therefore enable the proper quantitative staging of cachectic cancer patients.

CASCO is mainly based on the following constituents: (1) body weight loss and composition, (2) inflammation/metabolic disturbances/immunosuppression, (3) physical performance, (4) anorexia, and (5) quality of life (Argilés et al., 2011). Table 1 pictures components and measured parameters in more detail.

**Table 1:** **Components of CASCO**.

**Component**	**%**	**Measurement**	**Parameter**
Body weight loss and composition (BWC)	40	Body weight loss	
		Lean body mass	
Inflammation/metabolic disturbances/immunosupression (IMD)	20	Inflammation	Plasma CRP
			Plasma IL-6
		Metabolic disturbances	Plasma albumin
			Plasma pre-albumin
			Plasma lactate
			Plasma triglycerides
			Plasma urea
			Anaemia
			ROS plasma levels
			Glucose tolerance test/HOMA index altered
		Immunosupression	Absolute lymphocyte number
Physical performance (PHP)	15		Questionnaire of 5 questions related to physical activity.
Anorexia (ANO)	15		Questionnaire of 4 questions extracted from SNAQ of St. Louis VA Medical Centre.
Quality of life (QoL)	10		Questionnaire of 25 questions from QLQ-C30.

Body weight loss and composition (BWC) is essential to all definitions of cachexia. But, the fact that both loss of muscle and fat tissue coexist in the cachectic patient, stresses the importance of assessing any changes in relation to lean body mass. The second component of CASCO is inflammation/metabolic disturbances/immunosuppression (IMD). Inflammation is a key feature of the cachectic response (Fearon et al., 1999; Delano and Moldawer, 2006). It cannot be overlooked that there are also a number of metabolic disturbances present in many cachectic patients such as: glucose intolerance, anemia, and low levels of plasma albumin, most of them included in CASCO (see Table 1). Immunosuppression may also be an early marker of cachexia (Faber et al., 2009); therefore, assessment of the immune response could also be a valid indication for a cachexia staging system. The third component relates to physical performance (PHP). Even with a relative small decrease in muscle mass in cachexia, there may be a significant decrease in physical activity which are related to muscle performance (Fouladiun et al., 2007; Maddocks et al., 2010). Anorexia (ANO) constitutes the fourth parameter included in CASCO. Indeed, anorexia is present in cachexia in many diseases (Laviano et al., 2005). A decrease in food intake, by itself, promotes changes in quality of life and also conditions many metabolic alterations. Finally, the last component of CASCO is quality of life (QoL). Quality of life reflects not just changes in weight and physical performance but also in metabolic alterations (Fouladiun et al., 2007; Granda-Cameron et al., 2010). Therefore, it is an important point to consider.

These five different factors mentioned above, clearly interact between each other and represent the most important set of variables to assess the severity of the cachectic syndrome.

Bearing all this in mind, the aim of the present investigation was to clinically validate CASCO for its use in the classification of cachectic cancer patients. In addition, a simplified form of CASCO (miniCASCO) was designed to cope with the limitation of assessment methods and tools in some clinical centers.

## Patients and methods

### Patients

**Figure 1:**
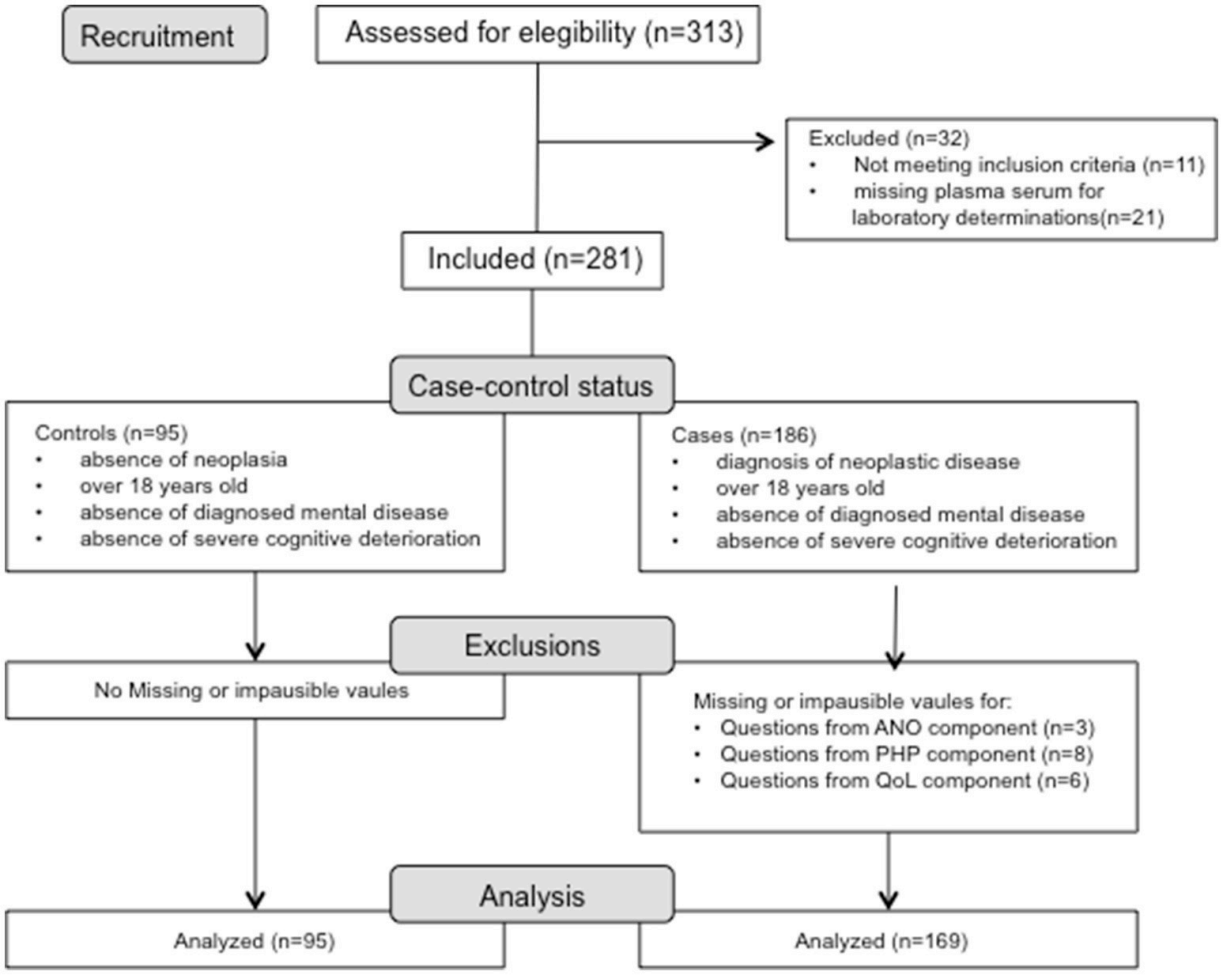
**Flow diagram of excluded participants in the CASCO Study with missing or implausible data**.

An observational prospective case-control study has been performed and a total of 186 carcinoma patients and 95 age-matched control subjects were included (see Participant flow chart in Figure 1). All the participants in the study were recruited at the Department of Medical Oncology (University of Cagliari, Cagliari, Italy) from June 2011 to September 2014. Inclusion criteria for the cancer patients were histologically confirmed cancer at any site, age≥18 years old, and the absence of diagnosed mental disease or severe cognitive deterioration. Inclusion criteria for the control subjects were absence of neoplasia, to be over 18 years old and absence of diagnosed mental disease or cognitive deterioration. Those patients affected by either non cancer-related nutritional alterations or inflammatory states leading to body weight loss were excluded from the study. All the enrolled patients and controls were evaluated for all parameters with the same methodology descripted below, reaching the CASCO score for all subjects. The clinical protocol was fully approved by the Ethics Committee of the University of Cagliari (Cagliari, Italy; control and patient subjects) and by Ethics Committee of the University of Barcelona (control subjects), and all patients and controls signed the approved written informed consent. Subject characteristics are presented in Tables 2, 3. Data were extracted, and the quality of the included studies was evaluated using the STROBE checklist.

**Table 2:** **Descriptive statistics for each group**.

**Group**	**Gender (%)**	**Age**	**Weight**	**Diagnosis**	**Ecog Ps**	**Stage**
Controls (*n* = 95)	males = 55%	56.34 ± 0.677 (95^*^)	76.73 ± 1.58 (93^*^)	Healthy subjects (*n* = 75)	0 (100%)	NV
	females = 45%			Patients suffering from non-neoplastic diseases^#^ (*n* = 20)	0 (100%)	NV
Oncologic patients (*n* = 186)	males = 59%	65.27 ± 0.877 (186^*^)	74.00 ± 1.92 (186^*^)	Carcinoma (*n* = 178)	0–1 (38%) 2–3 (62%)	I –IIIA 20% IIIB– IV 80%
	females = 41%			Mesothelioma, and sarcoma (*n* = 8)		

*Results are mean ± S.D and n (the number of patients and controls in parentheses*,

* *indicates the number depending of missing data)*.

# *asthma, hypertension, allergic rhinitis, muscle pain, high cholesterol levels*.

**Table 3:** **Carcinoma site diagnosed in cancer group**.

**Tumor site**	**n**	**Age**
Lung	32	70.81 (2.75)
Breast	27	59.78 (2.67)
Head and neck	21	63.19 (2.36)
Colon	17	69.16 (2.16)
Ovary	13	55 (3.79)
Pancreas	11	64.72 (3.87)
Prostate	10	76.3 (1.38)
Upper gastrointestinal	10	63 (3.29)
Rectum	8	68.71 (1.01)
Bile glands	7	69 (5.68)
Endometrium	4	64.5 (4.03)
Liver	3	64.66 (14.30)
Kidney	3	65.33 (5.66)
Other^*^	17	69.14 (2.49)

*Results are mean (S.D) and n the number of cancer patients*.

* *Other carcinoma sites: peritoneum, cervix, appendix, bladder; and other tumor types: lung sarcoma, pleura sarcoma, myelofibrosis, pleural mesothelioma and lung heteroplasia*.

### CASCO

The CAchexia SCOre was applied and calculated for each of the subjects as previously described (Argilés et al., 2011). The different elements of the score are shown in Table 1. More information related to the questionnaires and related calculations can be found in: http://hdl.handle.net/2445/65137 and http://www.ub.edu/cancerresearchgroup/.

### MCASCO

The simplified version of CASCO, miniCASCO (MCASCO), was applied and calculated for each of the subjects. The components of the MCASCO are shown in **Table 5**.

### Body weight loss and composition (BWC)

Body weight was measured at questionnaire time using an electronic balance (Health-o-Meter, Bridgeview, IL, USA), pre-illness weights being obtained by interview or by patient's data collection in clinical practices. Lean body mass (LBM) was assessed through different methods based on the planned instrumental oncological assessments. These included: (i) conventional bioelectrical impedance analysis (BIA) (Simons et al., 1995; Ellis, 2000) using a Bioelectric Impedance Analyser STA/BIA101 (Akern, Firenze, Italy) in 70% of the patients; (ii) dual-energy X-ray absorptiometry (DXA) (Plank, 2005; Ellegård et al., 2009) using a Hologic Delphi W scanner (Hologic Inc., Bedford, MA) in 10% of the patients; (iii) regional computed tomography (CT) scan analysis at L2-L3, currently considered the highest precision method, trough measurement by Tomovision Slice-o-Matic Software (Montreal, Canada) in 20% of the patients. It has to be pointed out that, although different approaches for the determination of body composition were used, good correlation have described between the different methodologies used (Fürstenberg and Davenport, 2011). LBM depletion was defined at baseline accordingly to standard international range for each method.

### Inflammation/metabolic disturbances/immunosuppression (IMD)

#### Inflammation

Peripheral venous blood samples were obtained from all patients and controls by venipuncture (BD Vacutainer, California, USA). The serum levels of CRP were measured by automatic centralized nephelometric analyser (AU640, Olympus, Germany), the results were expressed in mg/L. IL-6 were assessed by ELISA “sandwich” test, using monoclonal antibodies against specific molecular epitopes (DRG International, Springfield, NJ, USA by IAM Consulting, Parma, Italy). The assays were performed in semiautomatic ELISA analyser (DiaSorin Etilab, Guidonia, Italy) and the results expressed in pg/mL. The coefficients of variation for all these methods, in accordance with the quality control procedures, were always <5%.

#### Metabolic disturbances

Metabolic disturbances included the determination of albumin, pre-albumin, lactate, triglycerides, hemoglobin, urea, reactive oxygen species (ROS), and HOMA index. Albumin, pre-albumin, lactate, triglycerides, and urea levels were obtained during oncological clinical routine by hospital central laboratory (METROLAB 2300 (Wiener Lab) and the results expressed in mg/dL for pre-albumin, triglycerides, and urea and g/dL for albumin. HOMA index was calculated for each patient (Matthews et al., 1985). Determination of plasma levels of ROS were assessed by reactive oxygen species colorimetric assay (FORT test, Callegari SpA, Italy; Mantovani et al., 2003; Pavlatou et al., 2009). Haemoglobin levels were obtained carried out the routine blood count (Coulter LH750, Beckman-Coulter) and expressed in g/dL.

#### Immunosuppression

The immunosuppression was evaluated for each patient by absolute lymphocyte count, obtained from de routine blood count. Lymphocyte count has been well recognized as a valid marker of immune function as well as a prognostic marker (Bouwhuis et al., 2011). It is included in validated nutritional tools (such as the Mini Nutritional Assessment; Kabata et al., 2015; Bourdel-Marchasson et al., 2016). It has to be pointed out that the total lymphocyte count is not a fully convincing measure of immunosupression, although it is an “affordable” easy reliable measurement in a standard hospital. This, therefore, represent a minor limitation of the score particularly since it only represents 4% of CASCO.

### Physical performance (PHP)

In order to be able to assess the functional state of a cachectic patient, a physical performance questionnaire was used at the evaluation time. One question from EORTC QLQ-C30 (question number 1) was included. Its use is under permission of 1995 EORTC Quality of Life Group (Aaronson et al., 1993). The text of the questionnaire is: *During the past week: **1**. Have you noticed any particular decrease in the physical activities (i.e., at work, at home, at leisure etc…) that you normally carry out during the day?; **2**. Have you had any problems doing strenuous activities, like carrying a heavy shopping bag or a suitcase?; **3**. Have you noticed any loss of handgrip force?; **4**. Did you have to put more effort on climbing stairs?; **5**. Have you felt tired after walking approximately half a kilometer?*

### Anorexia (ANO)

Anorexia was estimated using a standard questionnaire [Simplified Nutrition Assessment Questionnaire (SNAQ)] (Wilson et al., 2005). Its use in CASCO is under permission of St. Louis GRECC Program of St. Louis VA Medical Centre. The text of the questionnaire is: *1. My appetite is (very poor, poor, average, good, very good); 2. When I eat (I feel full after eating only a few mouthfuls, I feel full after eating about a third of a meal, I feel full after eating over half a meal, I feel full after eating most of the meal, I hardly ever feel full); 3. Food tastes (very bad, bad, average, good, very good); 4. Normally I eat (less than one meal a day, one meal a day, two meals a day, three meals a day, more than three meals a day)*.

### Quality of life (QoL)

Concerning quality of life, CASCO includes 25 questions from EORTC QLQ-C30 (question numbers: 4–12, 14–17, 19–30). Questions related to physical performance or food intake were withdrawn. Its use in CASCO is under permission of 1995 EORTC Quality of Life Group (Aaronson et al., 1993). The text of the questionnaire is: *During the past week: 1. Do you need to stay in bed or a chair during the day?; 2. Do you need help with eating, dressing, washing yourself or using the toilet?; 3. Were you limited in doing either your work or other daily activities?; 4. Were you limited in pursuing your hobbies or other leisure time activities?; 5. Were you short of breath?; 6. Have you had pain?; 7. Did you need to rest?; 8. Have you had trouble sleeping?; 9. Have you felt weak?; 10. Have you felt nauseated?; 11. Have you vomited?; 12. Have you been constipated?; 13. Have you had diarrhea?; 14. Did pain interfere with your daily activities?; 15. Have you had difficulty in concentrating on things, like reading a newspaper or watching television?; 16. Did you feel tense?; 17. Did you worry?; 18. Did you feel irritable?; 19. Did you feel depressed?; 20. Have you had difficulty remembering things?; 21. Has your physical condition or medical treatment interfered with your family life?; 22. Has your physical condition or medical treatment interfered with your social activities?; 23. Has your physical condition or medical treatment caused you financial difficulties?; 24. How would you rate your overall health during the past week?; 25. How would you rate your overall quality of life during the past week?*

### Statistical analysis

The *Statistical Package for Social Sciences* (IBM v.21) was used to analyse the effects between groups. From a psychometric perspective, reliability, as an internal consistency parameter, was estimated using the Cronbach's α. In addition, Confirmatory Factorial Analysis (CFA) was conducted to estimate construct validity through EQS software (v6.0) and normative data were obtained from a classical point of view with position indexes. All the statistical techniques were carried out with a significance level of α = 0.05, correcting for reduction of type I error using the Bonferroni correction. Finally, cluster analysis between groups was performed to determine the breakpoints within the scale and estimate the maximum inertia centroids values. In addition, each statistical contrast includes the specification of sample size due to missing data presence.

## Results

### Analysis of reliability

The reliability coefficients were estimated using Cronbach's α for each of the general factors derived from the questionnaire (Table 4). The values obtained indicate high reliability for each of the factors studied

**Table 4:** **Reliability estimation through Conbrach's α**.

**Variable**	n^*^	**Cronbach's α**
Physical performance	276	0.928
Anorexia	279	0.793
Quality of life	275	0.945

*n, the number of patients and controls*,

* *indicates the number depending of missing data*.

### Construct validity

To estimate the validity of the construct, a model involving Confirmatory Factor Analysis (CFA) from the Correlation Matrix of Pearson was used. The value of the Maximum Likelihood Estimation (ML), applied to the matrix R, provided initial results in an adjusted model (χ^2^ = 675.11; *df* = 253, *p* < 0.001; Comparative Fit Index (CFI) = 0.912; Tucker Lewis Index (TLI) = 0.941; Root Mean Standard Error (RMSEA) = 0.04; Adjusted and Standardized Root Mean Residuals (SRMR) = 0.039; 95% Confidence Interval of SRMR 0.02–0.05; Ratio χ^2^/*df* = 3.277). All indicators, except for the statistical χ^2^, are compatible with a valid model, particularly the criterion derived from χ^2^/df. Additionally, it should be remarked that each of the latent factors assumes a significant proportion of the initial variance. Thus, PHP took 15% while ANO 18% and QoL 45%, the three representing 78% of the initial value, which is regarded as a high level of variance accounted for by the reduction of dimension.

### Discriminant validity

**Figure 2:**
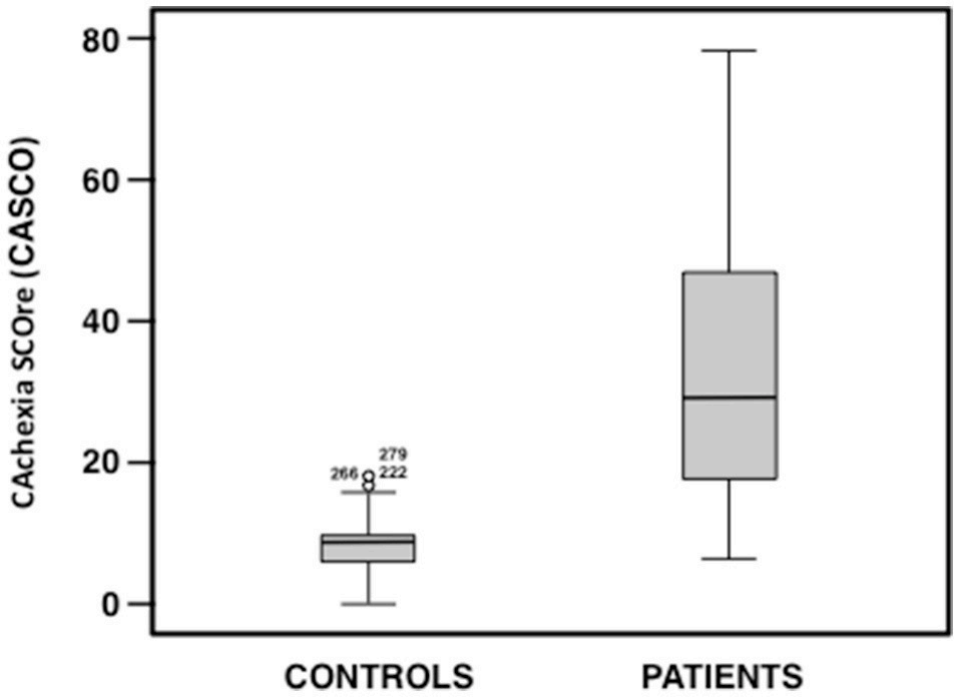
**Box plot of CASCO total score for each group (patient and control groups)**.

Using the CASCO score the two groups (patients and controls) were compared in order to estimate the discriminant validity of the score. The results depicted in Figure 2 show a very significant difference between groups (*t* = 145.77, *df* = 273, *p* < 0.001; *r* = 0.74; Confidence interval of mean difference at 95% between 14.01 and 29.86), indicating a high capacity in the discriminative ability of the total score. Thus, in the cancer group, the CASCO value was 32.54 with a standard deviation of 17.58, while in the control group it was 8.72 with a standard deviation of 3.56.

### Concurrent validity

**Figure 3:**
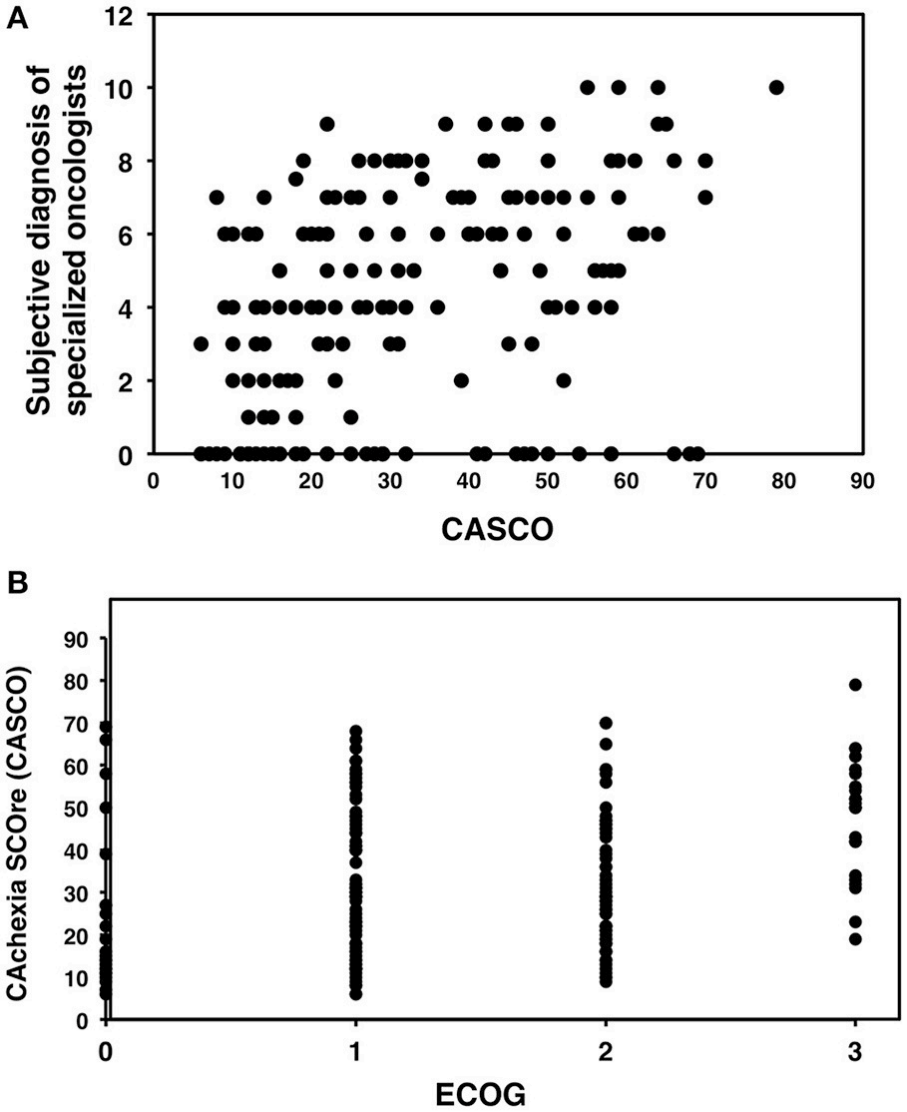
**Scatter plots of CASCO against other values of clinical assessment**. **(A)** Correlation between CASCO and the subjective diagnosis of specialized oncologists. **(B)** Correlation between CASCO and the Eastern Cooperative Oncology Group (ECOG) scale.

The correlation between the total CASCO values and those obtained using a subjective diagnosis of specialized oncologists (the Oncologist Team from the Department of Medical Oncology, University of Cagliari, Cagliari, Italy) was established. The subjective evaluation was based on the following question: “*Before applying CASCO, what is your perception of severity of patient's cachexia according to the following scale 0 (normal, absence of cachexia), 1, 2, 3, 4, 5, 6, 7, 8, 9, 10 (terminal, evident cachexia).”* Figure 3A shows the scatter plot between the two variables, characterized by estimating the Spearman correlation coefficient (*r*_*s*_ = 0.412, *p* < 0.001). The results indicate a clear positive relationship between the two variables and therefore, a high level of concurrent validity. Moreover, other external criteria were used: the Eastern Cooperative Oncology Group (ECOG) scale, which is a widely used score involved in assessing cancer patients. Figure 3B shows the scatter plot between the ECOG and CASCO scores. The Spearman correlation coefficient was: *r*_*s*_ = 0.290, *p* < 0.001, indicating a clear positive relationship between the two variables a moderate level of concurrent validity.

### Estimated classification

**Figure 4:**
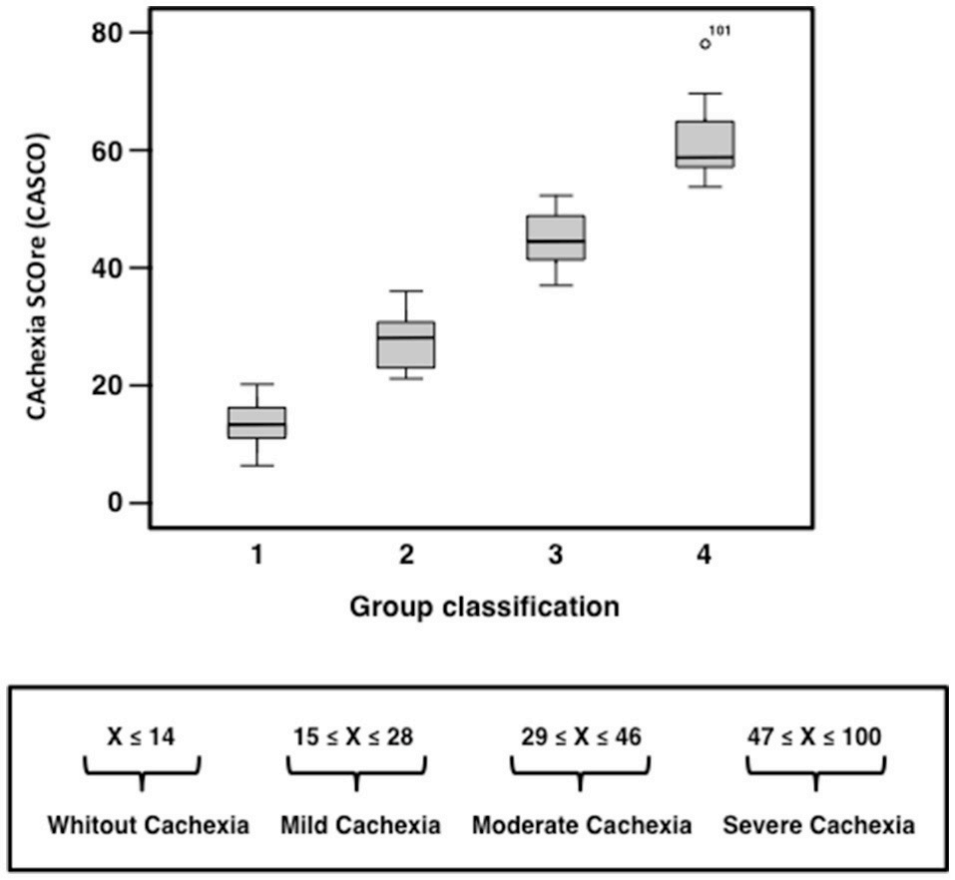
**CASCO distribution for each of the groups set for the diagnosis of the severity of cachexia**.

Using the CASCO values, three cut-off values were estimated by means of the application of a hierarchical cluster. Four groups were originally described, one exactly below the observed mean, and the other exactly over the mean; and the two last zones adjusted to every cue (inferior and superior). The three cut-off values were estimated through the maximization of the classification function using 95% confidence levels. This was accomplished by using a similarity matrix according to the metric properties of the variables and assuming multinormal distribution. The results show that the four groups were: no cachexia (≤14), mild cachexia (15–28), moderate cachexia (29–46) and finally, severe cachexia (>46). Figure 4 shows the distribution observed for each of the groups derived from the above criterion, indicating that the differences between groups were highly significant (*F* = 743.12, *df* = 3, 244; *p* < 0.001; ε = 0.61).

### MiniCASCO

**Figure 5:**
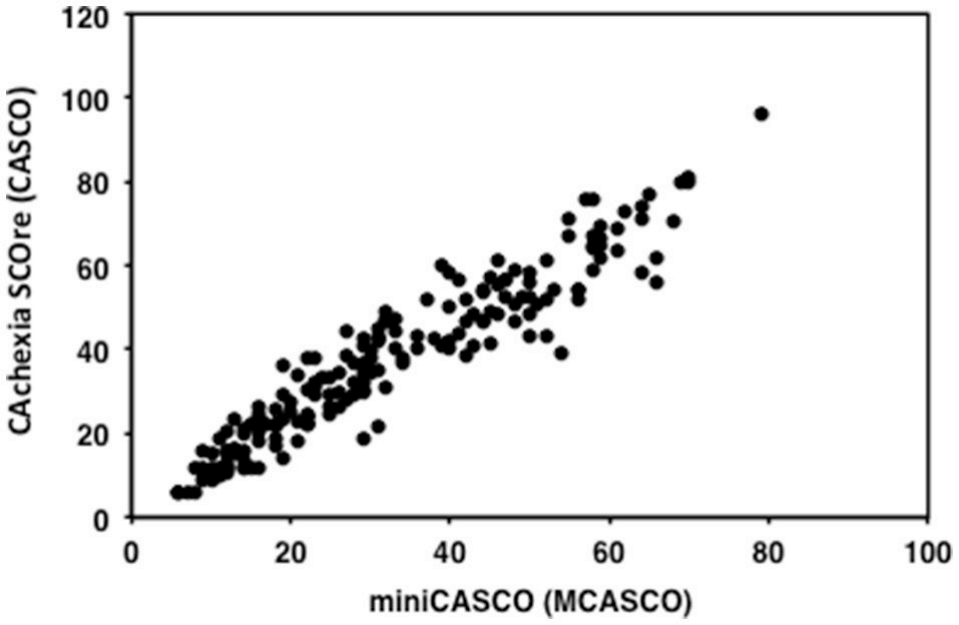
**Scatterplot of CASCO against MCASCO**.

A simplified version of CASCO, miniCASCO (MCASCO), was designed to avoid an excessive amount of clinical measurements which in some medical centers may be limiting. The components of the MCASCO are shown in Table 5. The correlation between the two values, i.e., the original CASCO and the reduced version MCASCO showed a highly significant coefficient (*r* = 0.964; *df* = 19.50; *p* < 0.001; Figure 5). This result ensures that the psychometric properties of CASCO are also present in the MCASCO test, therefore suggesting a feasible and quick assessment of the cachexia stage.

**Table 5:** **MiniCASCO**.

**Weight**	Body weight loss	
	Lean body mass	
Blood parameters	Plasma albumin	Metabolic disturbances
	Anaemia	
	CRP	Inflammation
	Absolute lymphocyte number	Immunosuppression
Questionnaires	Did you have to put more effort on climbing stairs?	Physical performance
	Have you felt tired after walking approximately half a kilometer?	
	My appetite is…	Anorexia
	When I eat…	
	Do you need to stay in bed or a chair during the day?	Quality of life
	Where you limited in doing either your work or other daily activities?	
	Were you limited in pursuing your hobbies or other leisure time activities?	
	Have you had pain?	
	Did you need to rest?	
	Did you feel weak?	
	Did pain interfere with your daily activities?	
	Have you had difficulty in concentrating on things, like reading a newspaper or watching television?	
	Has your physical condition or medical treatment interfered with your family life?	
	How would you rate your overall health during the past week?	

## Discussion

With the aim of validating the previously published CAchexia SCOre (CASCO) (Argilés et al., 2011), 186 cancer patients (males and females in a similar percentage) were recruited in this study (Table 2). As a reminder, one has to take into consideration that CASCO includes a combination of the following components: (1) body weight loss and composition, (2) inflammation/metabolic disturbances/immunosuppression, (3) physical performance, (4) anorexia, and (5) quality of life. CASCO was only slightly modified from the original published version (Argilés et al., 2011); thus absolute lymphocyte number was taken as a measure to evaluate immunosuppression (Table 1).

The study includes a heterogeneous cancer patient population. The most abundant type of cancer was lung carcinoma while kidney and liver cancer and other carcinoma sites included the smaller number of patients (see Table 3 for more information). Control subjects were either healthy (*n* = 75) or suffering from non-neoplastic diseases (asthma, hypertension, allergic rhinitis, muscle pain, high cholesterol levels; Table 2).

Interestingly, other cachexia classification studies also agree with the results obtained here (Fearon et al., 2011; Vigano et al., 2016). Indeed, Fearon et al. proposed a classification of cachectic patients based on identifying the following stages: precachexia, cachexia, and refractory cachexia (Fearon et al., 2011). The additional advantage of the classification proposed in the present study is that it involves a numerical scale and therefore can discriminate between patients in any of the three cachectic groups. Then, the study basically classifies patients into the following three groups that are associated with a numerical scoring: mild cachexia, moderate cachexia and severe cachexia. Conversely, other cachexia classifications do not provide any discrimination between patients in the same subgroup.

Although the obtained data seem to follow a logical pattern as compared with previous studies, we undertook a more rigorous validation based on correlating CASCO with other scores. From this point of view, we chose the ECOG (Oken et al., 1982) scale, which is a widely used score involved in assessing cancer patients. Although ECOG is not an specific scale fo cachexia, it has to be pointed out that it is widely used in cancer patients and also that there is no other quantitative cachexia scale at present to establish an alternative validation. Additionally, a subjective diagnosis of specialized oncologists was used (the oncologist team from the Department of Medical Oncology, University of Cagliari, Cagliari, Italy) and adequate statistically significant correlations between CASCO and the two scales were observed for both (only considering the patient values; Figures 3A,B).

In spite of the fact that this study clearly demonstrates that CASCO is a valid score in the clinical context, it can be argued that the large number of items (both measurements and questionnaires) could be a serious obstacle for its routinely use. Bearing this in mind, we developed a simplified version, the so-called MiniCASCO (MCASCO), containing only a reduced number of items (see Table 5). To reduce the number of items a component analysis was performed. The reduction of items was done based on factorial loadings of the items in the component and the discrimination index. This process was done for each component (PHP, ANO, and QoL). In addition to body weight loss and composition, blood measurements include only albumin, anemia, CRP, and absolute lymphocyte number (Table 5); together with a questionnaire containing two questions related with PHP, two related with ANO and 11 related with QoL (Table 5). It can be seen that there is an excellent correlation between CASCO and MCASCO (*r* = 0.964; Figure 5).

Altogether, the information presented here, first, serves to clinically validate CASCO for the staging of cachectic cancer patients and second, it provides a significantly plausible tool (MCASCO) to perform the staging in almost any clinical setting, since the majority of hospitals and clinics have access to the determination of the parameters included in MCASCO. It has to be pointed out that CASCO and MCASCO provide a new tool for the quantitative staging of cachectic cancer patients with a clear advantage over previous classifications (Fearon et al., 2011; Martin et al., 2015; Vigano et al., 2016).

## Author contributions

Each author has participated suficiently, intellectually or practically, in the work to take public responsibility for the content of the article, including the conception, design, and conduction of the experiment and for data interpretation (authorship). SB, AB, and RS carried out the studies, sample analysis, data analyses, performed the statistical analysis and helped to draft the manuscript. SB, RS, JA, CM participated in the design and coordination of the study, carried out the studies, and helped to draft the manuscript. JG, MP, FL helped to carry out the studies. JA, SB, and RS conceived the study, participated in the design, coordination of the study, drafted the manuscript, and revised it critically.

### Conflict of interest statement

The authors declare that the research was conducted in the absence of any commercial or financial relationships that could be construed as a potential conflict of interest.
